# Maximal exercise and erythrocyte epoxy fatty acids: *a lipidomics study*

**DOI:** 10.14814/phy2.14275

**Published:** 2019-11-28

**Authors:** Benjamin Gollasch, Guanlin Wu, Inci Dogan, Michael Rothe, Maik Gollasch, Friedrich C. Luft

**Affiliations:** ^1^ Experimental and Clinical Research Center (ECRC), a joint institution between the Charité University Medicine and Max Delbrück Center (MDC) for Molecular Medicine Berlin‐Buch Germany; ^2^ HELIOS Klinikum Berlin‐Buch Berlin Germany; ^3^ Max Delbrück Center for Molecular Medicine (MDC) in the Helmholtz Association Berlin Germany; ^4^ LIPIDOMIX GmbH Berlin Germany; ^5^ Nephrology/Intensive Care Section Charité Campus Virchow Berlin Germany

**Keywords:** eicosanoids, Exercise, lipidomics, red blood cells

## Abstract

Fatty acid (FA)‐derived lipid products generated by cytochrome P450 (CYP), lipoxygenase (LOX), and cyclo‐oxygenase (COX) influence cardiovascular function. However, plasma measurements invariably ignore 40% of the blood specimen, namely the erythrocytes. These red blood cells (RBCs) represent a cell mass of about 3 kg. RBCs are a potential reservoir for epoxy fatty acids, which on release could regulate vascular capacity. We tested the hypothesis that maximal physical activity would influence the epoxy fatty acid status in RBCs. We used a standardized maximal treadmill exercise according to Bruce to ensure a robust hemodynamic and metabolic response. Central hemodynamic monitoring was performed using blood pressure and heart rate measurements and maximal workload was assessed in metabolic equivalents (METs). We used tandem mass spectrometry (LC‐MS/MS) to measure epoxides derived from CYP monooxygenase, as well as metabolites derived from LOX, COX, and CYP hydroxylase pathways. Venous blood was obtained for RBC lipidomics. With the incremental exercise test, increases in the levels of various CYP epoxy‐mediators in RBCs, including epoxyoctadecenoic acids (9,10‐EpOME, 12,13‐EpOME), epoxyeicosatrienoic acids (5,6‐EET, 11,12‐EET, 14,15‐EET), and epoxydocosapentaenoic acids (16,17‐EDP, 19,20‐EDP) occurred, as heart rate, systolic blood pressure, and plasma lactate concentrations increased. Maximal (13.5 METs) exercise intensity had no effect on diols and various LOX, COX, and hydroxylase mediators. Our findings suggest that CYP epoxy‐metabolites could contribute to the cardiovascular response to maximal exercise.

## Abstract 2

Red blood cells (RBCs) are a potential reservoir for epoxy fatty acids, which on release could regulate vascular capacity. We tested the hypothesis that maximal physical activity would influence the epoxy fatty acid status in RBCs. In our study, we included all RBC FA lipid components in the circulating blood. Epoxides derived from CYP monooxygenase and metabolites derived from LOX, COX, and CYP hydroxylase pathways were measured in RBCs by LC–MS/MS tandem mass spectrometry.

## Graphical abstract

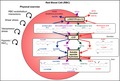

## Introduction

Optimal exercise performance requires an integrated organ system response. The entire cardiovascular system, pulmonary, systemic circulations enable increased gas exchange and oxygen availability to exercising muscles. The cardiovascular adaptations to maximal exercise include an increase in cardiac output resulting from an increased heart rate and to a lesser degree stroke volume, as well as a widening of the arteriovenous oxygen difference (Weiner, 1983). The rate of skeletal muscle blood flow degree and degree of oxygen consumption are increased (Weiner, 1983). The net effect of acute systemic hypoxia in quiescent skeletal muscle is vasodilation that occurs despite significant reflex increases in muscle sympathetic nerve activity. This vasodilation increases tissue perfusion and oxygen delivery to maintain tissue oxygen consumption (Dinenno, 2016). Although several mechanisms may be involved, there is evidence for local vasodilatory control mechanisms involving nitric oxide (NO) and prostaglandins (PGs) (Dinenno, 2016). When the stimulus is exacerbated *via* combined (sub)maximal exercise and systemic hypoxia to cause further red blood cell (RBC) deoxygenation, local vasodilation augments skeletal muscle blood flow. ATP release and nitrite reduction to NO have been shown to be involved in regulating this response (Dinenno, 2016; Zhang et al., 2018; Pernow et al., 2019).

**Figure 1:**
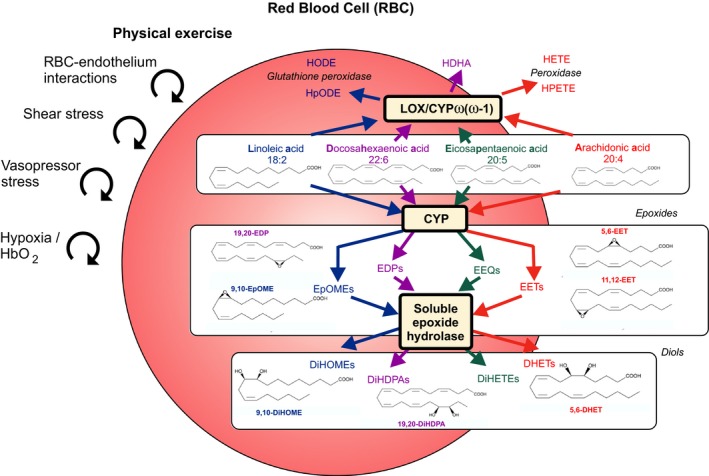
Short‐term maximal exercise associated with increased vasopressor and shear stress, red blood cell (RBC)‐endothelial interactions and tissue hypoxia affecting the content of epoxy‐metabolites in RBCs. The proposal illustrates cytochrome P450 epoxygenase (CYP) and 12‐ and 15‐lipoxygenase (LOX)/ CYP omega‐hydroxylase pathways. Linoleic (LA), arachidonic (AA), eicosapentaenoic (EPA), and docosahexaenoic acids (DHA) are converted to epoxyoctadecenoic acids (EpOMEs, e.g., 9,10‐EpOME), epoxyeicosatrienoic acid (EETs, e.g., 5,6‐EET, 11,12‐EET), epoxyeicosatetraenoic acids (EEQs) and epoxydocosapentaenoic acids (EDPs, e.g., 19,20‐EDP) by CYP, respectively. EpOMEs, EETs, EEQs and EDPs are metabolized to dihydroxyctadecenoic acids (DiHOMEs, e.g., 9,10‐DiHOME), dihydroxyeicosatrienoic acids (DHETs, e.g., 5,6‐DHET), dihydroxyeicosatetraenoic acids (DiHETEs) and dihydroxydocosapentaenoic acids (DiHDPAs, e.g., 19,20‐DiHDPA), respectively, by the soluble epoxide hydrolase (sEH) enzyme. LA, AA, EPA, and DHA are converted to hydroperoxylinoleic acids (HpODEs), hydroxyoctadecadienoic acids (HODEs), hydroxydocosahexaenoic acids (HDHAs), hydroperoxyeicosatetraenoic acids (HPETEs) and hydroxyeicosatetraenoic acids (HETEs) by LOX, CYP omega/(omega‐1)‐hydroxylase and peroxidase pathways. The metabolites measured within these pathways track the changes observed in LA, AA, EPA, and DHA, respectively. Arrows demarcate metabolic pathways evaluated.

PGs and other eicosanoids, such thromboxanes (TXs), leukotrienes (LTs), epoxides, and hydro(peroxy) fatty acids (or oxylipins) are lipid peroxidation products of 20‐carbon (eicosa‐) polyunsaturated fatty acids (PUFA). These products are generated by three separate enzyme families, namely cyclooxygenase (COX), lipoxygenase (LOX), and cytochrome P450 (CYP) expoygenases. These pathways catalyse lipid peroxidation in a highly regulated manner generating stereo and regio‐specific products (Fig. 1). Their expression is highly tissue‐localized and varies with inflammatory activation state. Primary products of COX, LOX, and CYP are metabolized, depending on cell type, into secondary eicosanoids and their metabolites, some of which are also potently bioactive (Fig. 1). The major metabolic pathways of PUFA epoxides are incorporation into phospholipids and hydrolysis to the corresponding PUFA diols by soluble epoxide hydrolase (sEH) (Spector and Kim, 2015). In a number of vascular beds, CYP‐derived epoxyeicosatrienoic acids (EETs) and other epoxides, such as 17,18‐epoxyeicosatetraenoic acid (17,18‐EEQ), can function as endothelium‐derived hyperpolarizing factors (EDHF) that contribute to vasodilation (Hu and Kim, 1993; Campbell et al., 1996; Hercule et al., 2007).

Recently, RBCs (~3 kg in human body) have been implicated to serve as a reservoir for epoxide fatty acids, in particular CYP‐derived EETs, which on release may act in a vasoregulatory capacity (Jiang et al., 2010; Jiang et al., 2011). Furthermore, sEH in the RBC and the increase in EETs resulting from its pharmacological inhibition presumably contributes to a greater degree on regional blood flows than sEH inhibition localized in the arterial wall (Yu et al., 2004; Jiang et al., 2011). Both *cis*‐ and *trans*‐EETs are stored in phospholipids and in RBCs. The greater vasodilator potency of *trans*‐ vs. *cis*‐EETs may contribute to the antihypertensive effects of sEH inhibitors in rats (Jiang et al., 2011). However, it is unknown whether physical exercise affects the RBC epoxy fatty acid status. We tested the hypothesis that acute, maximal physical exercise might affect RBC epoxy fatty acids. Maximal treadmill Bruce test was used to ensure sufficient and robust metabolic and hemodynamic response in healthy individuals. RBC lipidomics was performed using liquid chromatography‐mass spectrometry in tandem (LC–MS/MS).

## Methods

Prior to participation in the study, six healthy volunteers (5 male and 1 female; age 38 ± 15 years; body mass index 27.9 ± 6.6 kg/m^2^) signed informed consent forms which outlined the procedures to be taken and the possible risks involved. The study was approved by the Charité University Medicine institutional review board on the use of humans in research. All subjects were non‐trained and not taking medications. Recruitment was primarily *via* person‐to‐person interview. Following a routine physical examination at baseline levels each subject underwent a maximal treadmill Bruce test, which is recommended by guidelines for ergometry of the German Society of Cardiology (Bruce et al., 1973; Trappe and Lollgen, 2000). The test was preceded by 2 x 3 min warm up periods (stages 1 and 2 of the Bruce protocol) during which treadmill speed was maintained at a constant speed of 2.7 km/h and at zero or 5 per cent grade (Table 1) (Gollasch et al., 2019). Treadmill speed and grade were then increased at three min intervals. The test was terminated when the subjects informed the investigator that they could no longer proceed. Workload was assessed in metabolic equivalents (METs) (Table 1).

**Table 1:** Bruce protocol and estimated metabolic equivalents of task (METs).

Stages	Duration (in min)	Grade (in %)	Speed (in km/h)	Speed (in MPH)	METs
1	3	0	2.7	1.7	Warm up
2	3	5	2.7	1.7	Warm up
3	3	10	2.7	1.7	5
4	3	12	4.0	2.5	7
5	3	14	5.4	3.4	10
6	3	16	6.7	4.2	13
7	3	18	8.0	5.0	15
8	3	20	8.8	5.5	18

**Figure 2:**
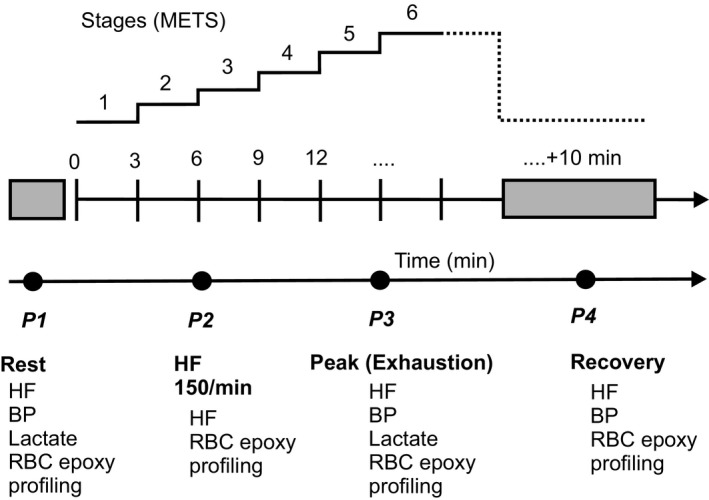
Following abbreviations are: HF, heart rate; BP, blood pressure; RBC, red blood cell; epoxy, epoxide fatty acids; METs, metabolic equivalents of task.

Heart rates were monitored continuously by heart‐rate monitor worn around the subject’s torso (Polar T31, Polar Electro, Kempele, Finland). Arterial pressure was measured *via* a sphygmomanometer (Critikon, Inc., Johnson & Johnson, New Jersey, USA), which comprised an inflatable (Riva‐Rocci) cuff placed around the upper arm. Venous blood was collected from a catheter placed in a contralateral forearm vein (i.e., the antecubital vein) of each subject in the sitting position prior to the exercise test (−10 mins, *P1*), after termination of the test (exhaustion, *P3*), and 10 min after the end of the running test (recovery period, *P4*) (Fig. 2). An additional blood sample was collected in each subject during running when the heart rate reached 150 beats per minute (*P2*). All samples were analyzed for total and free RBC epoxide lipid mediator status. RBCs were separated from EDTA blood by centrifugation and metabolites were determined by liquid chromatography tandem mass (LC–MS/MS) spectrometry as described in Fischer et al., (2014). Serum lactate was determined in blood samples obtained from ear lobe at rest and at maximal workload (Fig. 2).

Descriptive statistics were calculated and variables were examined for meeting assumptions of normal distribution without skewness and kurtosis. In order to determine statistical significance between the trials at the various time intervals, one‐way repeated measures analysis of variance (ANOVA) was conducted and the 0.05 level of significance (*P*) was chosen. The analysis included Mauchly's test of sphericity followed by applying the test of within‐subjects’ effects with Greenhouse–Geisser correction, which is used for repeated‐measures ANOVA when the assumption of sphericity is violated. When significant differences were found, Tukey’s honestly significant difference post hoc test was used for pairwise comparisons. The nonparametric test (distribution‐free) Friedman’s test was used when the normal distribution was violated. Planned hypotheses (one‐tailed or two‐tailed paired *t*‐tests as appropriate) were tested to follow up the initial ANOVA findings. All data are presented as mean ± SD. All statistical analyses were performed using SPSS Statistics software (IBM Corporation, Armonk, NY, USA) or All‐Therapy statistics beta (AICBT Ltd, Vancouver, Canada).

## Results

### Hemodynamic and metabolic response

Exercise significantly increased heart rate, systolic, and diastolic blood pressure (Table 2). Data prior to the exercise test (−10 mins, *P1*), after termination of the test (exhaustion, *P3*), and 10 min after the end of the running test (recovery period, *P4*) are shown in Figure 2. An additional blood sample (*P2*) was obtained throughout exercise when the heart rate reached 150 beats per minute. After exercise, heart rate and systolic blood pressure returned to resting levels (*P1)* in the recovery period (*P4*) (Table 2 **,** *P *> 0.05, Tukey’s post hoc test) (Gollasch et al., 2019). All participants terminated exercise at a maximal workload of 13.50 ± 1.97 METs (Bruce stage, 6.33 ± 0.82). Exercise increased lactate concentrations from 1.3 + 0.3 (*P1*) to 9.5 ± 2.1 mmol/L (*P3*) (*P* < 0.0001, *t*‐test).

**Table 2:** Effects of exercise on hemodynamics (means ± SD, *n* = 6).

Parameter	Time point 1, *P1* (rest)	Time point 2, *P2* (HF 150)	Time point 3, *P3* (exhaustion)	Time point 4, *P4* (recovery)	Greenhouse–Geisser, *P* value
Heart rate (beats per min)	71 ± 10	150	185 ± 6	94 ± 11	<0.001
Systolic arterial blood pressure (mm Hg)	135.3 ± 9.1	n.d.	190.3 ± 16.6	127.5 ± 13.1	<0.001
Diastolic arterial blood pressure	81.2 ± 14.4	n.d.	90.7 ± 16.4	76.3 ± 10.2	0.097

n.d., not determined

### Fatty acid‐derived *cis*‐mediators

Since the impact of acute exercise on individual RBC epoxide fatty acid status is unknown, we initially used an explorative statistical approach and measured the total *cis‐*metabolites in RBCs at: baseline (rest, *P1*), the time when heart rate reached 150 beats per min (*P2*), exhaustion (*P3*), and recovery (*P4*) (Table 3). Our ANOVA analysis indicated differences in the total levels of epoxyoctadecenoic acids (9,10‐EpOME, 12,13‐EpOME), epoxyeicosatrienoic acids (5,6‐EET, 11,12‐EET, 14,15‐EET), and epoxydocosapentaenoic acids (16,17‐EDP, 19,20‐EDP) (Table 3). However, we did not observe differences in the total levels of epoxyeicosatetraenoic acids (5,6‐EEQ, 8,9‐EEQ, 11,12‐EEQ, 14,15‐EEQ), 8,9‐EET, 10,11‐EDP, 13,14‐EDP, hydroxyeicosapentaenoic acids (5‐HEPE, 8‐HEPE, 9‐HEPE, 12‐HEPE, 15‐HEPE, 18‐HEPE) and hydroxyeicosatetraenoic acids (5‐HETE, 8‐HETE, 9‐HETE, 11‐HETE, 12‐HETE, 15‐HETE). We identified no differences in hydroxydocosahexaenoic acids (4‐HDHA, 7‐HDHA, 8‐HDHA, 10‐HDHA, 11‐HDHA, 13‐HDHA, 14‐HDHA, 16‐HDHA, 17‐HDHA, 20‐HDHA) and 13‐HODE levels. Of note, RBCs did not contain relevant levels of 19‐HEPE, 20‐HEPE, 19‐HETE, and 20‐HETE (detection threshold, <0.05 ng/g) (Table 3). The data demonstrate an accumulation of total 5,6‐EET, 11,12‐EET, 14,15‐EET, 16,17‐EDP, 19,20‐EDP 9,10‐EpOME, and 12,13‐EpOME concentrations in RBCs at exhaustion (for all *P* < 0.05, *t*‐tests), which are potent vasodilators (Siegfried et al., 1990; Jiang et al., 2010; Jiang et al., 2011; Morin et al., 2011). Accumulation of total 5,6‐EET, 11,12‐EET, 14,15‐EET, 16,17‐EDP, 19,20‐EDP, 9,10‐EpOME and 12,13‐EpOME levels in RBCs persisted in the recovery period (10 min) (*P* < 0.05, *t*‐tests). The data suggest that exercise leads to an accumulation of various CYP (i.e. *cis*) epoxy‐metabolites into membrane‐bound compartments of RBCs under exhaustive exercise or in ischemia in healthy individuals, which on release may act in a vasoregulatory capacity.

**Table 3:** Total *cis*‐metabolites in erythrocytes in response to exhaustive exercise (*n* = 6).

Metabolite (ng/g)	Time point 1 (rest), *P1*	Time point 2 (HF 150),* P2*	Time point 3 (exhaustion),* P3*	Time point 4 (recovery),* P4*	Greenhouse–Geisser, *P* value
13‐HODE	1258.0 ± 382.54	1124.22 ± 214.01	1296.36 ± 330.21	1329.40 ± 329.97	0.550
9,10‐EpOME	101.62 ± 11.50	122.80 ± 13.14	125.09 ± 16.78	128.38 ± 17.81	Friedman *P* = 0.002
12,13‐EpOME	99.25 ± 12.74	119.61 ± 15.71	125.09 ± 18.65	129.29 ± 22.75	0.001
9,10‐DiHOME	3.34 ± 1.50	6.30 ± 5.59	3.48 ± 1.00	4.68 ± 2.99	Friedman *P* = 0.108
12,13‐DiHOME	7.10 ± 2.80	11.06 ± 7.50	7.68 ± 2.06	9.97 ± 5.00	Friedman *P* = 0.056
5,6‐EET	54.75 ± 6.93	64.05 ± 5.61	61.67 ± 9.06	63.80 ± 8.11	0.021
8,9‐EET	70.70 ± 11.08	77.28 ± 7.25	75.20 ± 12.31	79.33 ± 11.92	0.247
11,12‐EET	71.69 ± 9.72	83.11 ± 9.75	80.74 ± 13.51	85.32 ± 12.54	0.039
14,15‐EET	79.97 + 13.23	93.61 ± 12.03	89.06 ± 14.96	94.77 ± 14.49	0.038
5,6‐DHET	4.67 ± 0.46	5.19 ± 0.51	4.77 ± 0.15	5.14 ± 0.63	0.191
8,9‐DHET	3.29 ± 0.44	3.74 ± 0.37	3.57 ± 0.46	3.74 ± 0.51	0.202
11,12‐DHET	1.97 ± 0.36	2.28 ± 0.25	2.11 ± 0.21	2.18 ± 0.30	0.279
14,15‐DHET	1.68 ± 0.32	1.75 ± 0.15	1.71 ± 0.13	1.79 ± 0.24	0.790
5,6‐EEQ	1.14 ± 0.63	1.23 ± 0.58	1.09 ± 0.46	1.17 ± 0.60	0.10
8,9‐EEQ	3.00 ± 1.15	3.03 ± 0.75	3.26 ± 0.93	3.22 ± 1.11	0.673
11,12‐EEQ	2.38 ± 0.78	2.48 ± 0.65	2.24 ± 0.59	2.57 ± 0.82	0.433
14,15‐EEQ	2.67 ± 0.86	2.75 ± 0.62	2.87 ± 0.66	2.91 ± 0.80	0.583
17,18‐EEQ	3.62 ± 0.94	3.69 ± 0.98	3.62 ± 0.78	3.64 ± 1.02	0.988
5,6‐DiHETE	0.78 ± 0.44	0.45 ± 0.40	0.72 ± 0.61	0.16 ± 0.38	0.090
8,9‐DiHETE	0	0	0	0	n/a
11,12‐DiHETE	0	0	0	0	n/a
14,15‐DiHETE	0	0	0	0	n/a
17,18‐DiHETE	3.49 ± 1.33	3.95 ± 0.99	4.50 ± 0.83	3.82 ± 0.64	0.153
7,8‐EDP	29.72 ± 5.06	33.36 ± 6.52	31.70 ± 7.26	33.30 ± 7.33	0.175
10,11‐EDP	42.64 ± 7.31	48.27 ± 10.14	45.13 ± 9.83	48.17 ± 11.21	0.122
13,14‐EDP	21.34 ± 4.31	25.22 ± 6.70	23.56 ± 5.30	25.42 ± 6.01	0.096
16,17‐EDP	27.04 ± 3.95	32.08 ± 6.97	30.73 ± 6.33	32.70 ± 7.22	0.021
19,20‐EDP	26.50 ± 4.83	30.84 ± 7.20	29.00 ± 6.00	31.32 ± 6.45	0.015
7,8‐DiHDPA	0.79 ± 0.31	0.78 ± 0.27	0.83 ± 0.19	0.98 ± 0.19	0.157
10,11‐DiHDPA	0.17 ± 0.10	0.18 ± 0.10	0.19 ± 0.11	0.18 ± 0.09	0.671
13,14‐DiHDPA	0.23 ± 0.18	0.24 ± 0.19	0.22 ± 0.17	0.23 ± 0.18	Friedman *P* = 0.800
16,17‐DiHDPA	0.25 ± 0.30	0.27 ± 0.31	0.38 ± 0.31	0.34 ± 0.29	0.501
19,20‐DiHDPA	0.05 ± 0.13	0.07 ± 0.16	0.07 ± 0.16	0.00 ± 0.00	Friedman *P* = 1.000
5‐HETE	214.23 ± 135.63	143.99 ± 39.23	189.26 ± 66.21	190.40 ± 44.62	0.382
8‐HETE	96.31 ± 52.88	72.09 ± 17.92	88.21 ± 24.46	89.10 ± 18.74	0.428
9‐HETE	116.82 ± 54.61	91.29 ± 21.93	106.86 ± 30.34	113.23 ± 22.46	0.430
11‐HETE	249.20 ± 130.33	181.35 ± 40.77	225.53 ± 59.37	248.41 ± 56.47	0.353
12‐HETE	118.25 ± 54.03	90.30 ± 20.67	114.03 ± 37.09	112.58 ± 22.98	0.384
15‐HETE	114.64 ± 70.53	81.36 ± 22.06	106.69 ± 34.94	105.07 ± 28.06	0.426
19‐HETE	0	0	0	0	n/a
20‐HETE	0	0	0	0	n/a
5‐HEPE	8.61 ± 5.43	5.92 ± 1.53	7.59 ± 2.24	8.09 ± 2.18	0.364
8‐HEPE	3.25 ± 1.95	2.18 ± 0.33	2.98 ± 0.98	2.86 ± 0.98	0.365
9‐HEPE	4.70 ± 2.52	3.81 ± 1.29	4.54 ± 1.27	4.53 ± 1.53	0.449
12‐HEPE	5.09 ± 2.33	4.17 ± 1.29	4.75 ± 1.70	5.14 ± 1.92	0.508
15‐HEPE	4.93 ± 2.27	4.14 ± 0.66	4.29 ± 1.02	4.48 ± 0.88	Friedman *P* = 0.772
18‐HEPE	15.62 ± 8.93	12.08 ± 4.44	13.69 ± 3.72	14.21 ± 3.70	0.496
19‐HEPE	0	0	0	0	n/a
20‐HEPE	0	0	0	0	n/a
10,17‐DiHDHA	0	0	0	0	n/a
4‐HDHA	116.85 ± 52.93	81.07 ± 11.71	111.98 ± 31.57	111.76 ± 27.96	0.343
7‐HDHA	28.35 ± 8.70	23.03 ± 3.32	27.20 ± 5.92	29.38 ± 6.92	0.358
8‐HDHA	48.33 ± 12.77	36.50 ± 5.34	47.08 ± 12.37	49.39 ± 12.78	0.249
10‐HDHA	34.29 ± 12.83	25.41 ± 3.45	32.34 ± 8.87	34.11 ± 9.39	0.358
11‐HDHA	29.71 ± 10.19	23.66 ± 4.09	30.18 ± 9.39	30.88 ± 8.57	0.448
13‐HDHA	48.34 ± 12.09	39.15 ± 5.08	48.70 ± 13.74	49.00 ± 11.68	0.350
14‐HDHA	60.42 ± 14.68	49.70 ± 7.96	61.95 ± 18.41	62.66 ± 17.61	0.404
16‐HDHA	44.51 ± 11.95	35.68 ± 4.31	43.14 ± 11.49	44.97 ± 11.68	0.382
17‐HDHA	75.87 ± 22.48	58.99 ± 6.36	79.46 ± 17.22	76.49 ± 21.83	0.283
20‐HDHA	65.09 ± 21.77	47.56 ± 6.85	63.43 ± 16.05	16.05 ± 17.91	0.285
21‐HDHA	0	0	0	0	n/a
22‐HDHA	0	0	0	0	n/a

To provide possible insights into the nature of this accumulation, we measured free epoxy and hydroxy metabolites in RBCs. We detected significant levels of free 13‐HODE, 9,10‐EpOME, 12,13‐EpOME, 9,10‐DiHOME, 12,13‐DiHOME, 14,15‐DHET, 12‐HETE, 12‐HEPE, 4‐HDHA, 14‐HDHA, and 16‐HDHA in RBCs (i.e., above the detection level, cf. methods), although no changes occurred in response to exercise (Table 4). Exercise did not induce significant levels of the majority of free fatty acid metabolites and PGs, including 5,6‐EET, 8,9‐EET, 11,12‐EET, 14,15‐EET, 5,6‐DHET, 8,9‐DHET, 5,6‐EEQ, 8,9‐EEQ, 11,12‐EEQ, 14,15‐EEQ, 17,18‐EEQ, 8,9‐DiHETE, 11,12‐DiHETE, 14,15‐DiHETE, 17,18‐DiHETE, 7,8‐EDP, 10,11‐EDP, 13,14‐EDP, 16,17‐EDP, 19,20‐EDP, 7,8‐DiHDPA, 10,11‐DiHDPA, 13,14‐DiHDPA, 16,17‐DiHDPA, 19,20‐DiHDPA, LTB4, LXA4, 5‐HETE, 8‐HETE, 9‐HETE, 11‐HETE, 15‐HETE, 19‐HETE, 20‐HETE, 5‐HEPE, 8‐HEPE, 15‐HEPE, 18‐HEPE, 19‐HEPE, 20‐HEPE, 10,17‐DiHDHA, 7‐HDHA, 8‐HDHA, 10‐HDHA, 11‐HDHA, 13‐HDHA, 17‐HDHA, 20‐HDHA, 21‐HDHA, 22‐HDHA, PGH2, PGE2, 15‐keto‐PGE2, 13,14‐dihydro‐15‐keto‐PGE2, Bicyclo PGE2 (a), Bicyclo PGE2 (b), PGD2, PGI2, 15‐deoxy‐delta 12,14‐PGI2, PGF2a, TXB2, 11‐dehydro TXB2, 6‐keto‐PGF1a, PGE3, TXB3, 11‐dehydro TXB3, d17‐6‐keto‐PGF1a (Table 4; detection level of 0.05 ng/g, each). The data suggest that maximal exercise does not result in accumulation of free epoxy‐metabolites and other fatty acid metabolites in RBCs for their uptake into membrane‐bound compartments.

**Table 4:** Free *cis‐*metabolites in red blood cells (RBCs) in response to exhaustive exercise (*n* = 6).

Metabolite (ng/g)	Time point 1 (rest),* P1*	Time point 2 (HF 150),* P2*	Time point 3 (exhaustion),* P3*	Time point 4 (recovery),* P4*	Greenhouse–Geisser, *P* value
13‐HODE	63.81 ± 27.94	58.56 ± 3.82	53.29 ± 5.15	53.36 ± 8.77	0.474
9,10‐EpOME	9.05 ± 1.17	1.17 ± 1.23	8.31 ± 0.82	8.34 ± 1.17	0.377
12,13‐EpOME	9.17 ± 1.39	9.70 ± 0.95	8.97 ± 0.92	9.06 ± 0.93	0.612
9,10‐DiHOME	1.87 ± 0.17	2.12 ± 0.43	1.90 ± 0.23	1.85 ± 0.22	Friedman *P* = 0.512
12,13‐DiHOME	4.18 ± 0.43	4.63 ± 0.51	4.09 ± 0.44	4.46 ± 0.18	0.076
5,6‐EET	0	0	0	0	n/a
8,9‐EET	0	0	0	0	n/a
11,12‐EET	0	0	0	0	n/a
14,15‐EET	0	0	0	0	n/a
5,6‐DHET	0	0	0	0	n/a
8,9‐DHET	0	0	0	0	n/a
14,15‐DHET	0.81 ± 0.40	0.85 ± 0.43	1.04 ± 0.05	0.84 ± 0.41	Friedman *P* = 0.398
5,6‐EEQ	0	0	0	0	n/a
8,9‐EEQ	0	0	0	0	n/a
11,12‐EEQ	0	0	0	0	n/a
14,15‐EEQ	0	0	0	0	n/a
17,18‐EEQ	0	0	0	0	n/a
8,9‐DiHETE	0	0	0	0	n/a
11,12‐DiHETE	0	0	0	0	n/a
14,15‐DiHETE	0	0	0	0	n/a
17,18‐DiHETE	0	0	0	0	n/a
7,8‐EDP	0.05 ± 0.14	0	0	0	n/a
10,11‐EDP	0	0	0	0	n/a
13,14‐EDP	0	0	0	0	n/a
16,17‐EDP	0	0	0	0	n/a
19,20‐EDP	0	0	0	0	n/a
7,8‐DiHDPA	0	0	0	0	n/a
10,11‐DiHDPA	0	0	0	0	n/a
13,14‐DiHDPA	0	0	0	0	n/a
16,17‐DiHDPA	0	0	0	0	n/a
19,20‐DiHDPA	0	0	0	0	n/a
LTB4	0	0	0	0	n/a
LXA4	0	0	0	0	n/a
5‐HETE	0	0	0	0	n/a
8‐HETE	0	0	0	0	n/a
9‐HETE	0	0	0	0	n/a
11‐HETE	0	0	0	0	n/a
12‐HETE	10.25 ± 2.84	12.26 ± 4.22	14.83 ± 7.32	13.77 ± 6.74	0.278
15‐HETE	0	0	0	0	n/a
19‐HETE	0	0	0	0	n/a
20‐HETE	0	0	0	0	n/a
5‐HEPE	0	0	0	0	n/a
8‐HEPE	0	0	0	0	n/a
12‐HEPE	1.34 ± 0.58	1.74 ± 0.78	1.88 ± 1.20	1.91 ± 1.02	Friedman *P* = 0.073
15‐HEPE	0	0	0	0	n/a
18‐HEPE	0	0	0	0	n/a
19‐HEPE	0	0	0	0	n/a
20‐HEPE	0	0	0	0	n/a
10,17‐DiHDHA	0	0	0	0	n/a
4‐HDHA	2.82 ± 1.04	3.12 ± 1.03	2.83 ± 0.75	2.87 ± 0.67	0.488
7‐HDHA	0	0	0	0	n/a
8‐HDHA	0	0	0	0	n/a
10‐HDHA	0	0	0	0	n/a
11‐HDHA	0	0	0	0	n/a
13‐HDHA	0	0	0	0	n/a
14‐HDHA	2.35 ± 1.44	2.16 ± 1.19	2.16 ± 0.97	2.77 ± 1.48	0.341
16‐HDHA	0.13 ± 0.21	0.29 ± 0.37	0.24 ± 0.26	0.18 ± 0.21	Friedman *P* = 0.446
17‐HDHA	0	0	0	0	n/a
20‐HDHA	0	0	0	0	n/a
21‐HDHA	0	0	0	0	n/a
22‐HDHA	0	0	0	0	n/a
PGH2	0	0	0	0	n/a
PGE2	0	0	0	0	n/a
15‐keto‐PGE2	0	0	0	0	n/a
13,14‐dihydro‐15‐keto‐PGE2	0	0	0	0	n/a
Bicyclo PGE2 (a)	0	0	0	0	n/a
Bicyclo PGE2 (b)	0	0	0	0	n/a
PGD2	0	0	0	0	n/a
PGI2	0	0	0	0	n/a
15‐deoxy‐delta 12,14‐PGI2	0	0	0	0	n/a
PGF2a	0	0	0	0	n/a
TXB2	0	0	0	0	n/a
11‐dehydro TXB2	0	0	0	0	n/a
6‐keto‐PGF1a	0	0	0	0	n/a
PGE3	0	0	0	0	n/a
TXB3	0	0	0	0	n/a
11‐dehydro TXB3	0	0	0	0	n/a
d17‐6‐keto‐PGF1a	0	0	0	0	n/a

### Diols

The main pathway of EET, EpOME, EEQ, and EDP metabolism is conversion to DHETs, DiHOMEs, dihydroxyeicosatetraenoic acids (DiHETEs), and dihydroxydocosapentaenoic acids (DiHDPAs) by the sEH, respectively (Fig. 1) (Spector et al., 2004). Since acute exercise might have caused EET, EpOME, EEQ, and EDP accumulation rapidly degraded to their diols, we analyzed their individual levels (Table 3) and the sums of the individual break‐down products and their respective diols (Table 5). Exercise did not change the levels of 9,10‐DiHOME, 12,13‐DiHOME, 5,6‐DHET, 8,9‐DHET, 11,12‐DHET, 14,15‐DHET, 5,6‐DiHETE, 17,18‐DiHETE, 7,8‐DiHDPA, 10,11‐ DiHDPA, 13,14‐DiHDPA, 16,17‐DiHDPA, 19,20‐DiHDPA (Table 3), but increased the levels 9,10‐EpOME, 12,13‐EpOME, 5,6‐EET, 11,12‐EET, 14,15‐EET, 16,17‐EDP, and 19,20‐EDP plus their respective diols (Table 5).

**Table 5:** Concentrations of individual total *cis‐*epoxides plus their respective diols in erythrocytes in response to exhaustive exercise (*n* = 6).

Epoxides or Diols (ng/g)	Time point 1 (rest), *P1*	Time point 2 (HF 150),* P2*	Time point 3 (exhaustion),* P3*	Time point 4 (recovery),* P4*	Greenhouse–Geisser, *P* value
9,10‐ EpOME + 9,10‐DiHOME	104.97 ± 12.95	129.09 + 15.44	128.57 ± 17.62	133.06 ± 18.67	<0.001
12,13‐EpOME+12,13‐DiHOME	106.35 ± 15.49	130.68 ± 19.66	132.77 ± 20.26	139.26 ± 26.13	<0.001
5,6‐EET+5,6‐DHET	59.42 ± 6.92	69.24 ± 5.40	66.45 ± 9.00	68.94 ± 8.52	0.012
8,9‐EET+8,9‐DHET	73.99 ± 11.02	81.03 ± 7.10	78.78 ± 12.05	83.07 ± 11.91	0.217
11,12 EET+11,12‐DHET	73.68 ± 9.71	85.39 ± 9.65	82.85 ± 13.42	87.50 ± 12.57	0.035
14,15‐EET+14,15‐DHET	81.65 ± 13.31	95.37 ± 12.07	90.76 ± 14.88	96.56 ± 14.54	0.036
5,6‐EEQ+5,6‐DiHETE	1.92 ± 0.84	1.69 ± 0.71	1.82 ± 0.91	1.33 ± 0.56	0.376
8,9‐EEQ+8,9‐DiHETE	3.00 ± 1.15	3.04 ± 0.75	3.26 ± 0.94	3.22 ± 1.11	0.673
11,12‐EEQ+11,12‐DiHETE	2.39 ± 0.78	2.49 ± 0.64	2.24 ± 0.59	2.57 ± 0.83	0.435
14,15‐EEQ+14,15‐DiHETE	2.67 ± 0.86	2.75 ± 0.62	2.87 ± 0.66	2.91 ± 0.80	0.589
17,18‐EEQ+17,18‐DiHETE	7.11 ± 1.80	7.64 ± 0.88	8.13 ± 1.18	7.46 ± 1.54	0.174
7,8‐EDP+7,8‐DiHDPA	30.52 ± 5.35	34.15 ± 6.69	32.53 ± 7.36	34.28 ± 7.36	0.170
10,11‐EDP+10,11‐DiHDPA	42.81 ± 7.39	48.46 ± 10.19	45.32 ± 9.89	48.35 ± 11.27	0.120
13,14‐EDP+13,14‐DiHDPA	21.57 ± 4.44	25.46 ± 6.85	23.78 ± 5.47	25.65 ± 6.19	0.096
16,17‐EDP+16,17‐DiHDPA	27.29 ± 4.07	32.36 ± 7.06	31.12 ± 6.61	33.05 ± 7.51	0.020
19,20‐EDP+19,20‐DiHDPA	26.55 ± 4.84	30.90 ± 7.20	29.07 ± 6.10	31.32 ± 6.45	0.016

### Diol/epoxide ratios

We next calculated diol/epoxide ratios in RBCs and analyzed their changes in response to exhaustive exercise and post exercise (Table 6). Our ANOVA analysis revealed no alterations in ratios for each substrate class in RBCs *in vivo* during and post exercise. Similar results were found for the individual metabolites (Table 7). Furthermore, we detected that the four classes of epoxy‐metabolites are unequally hydrolyzed to appear in the RBCs (Greenhouse‐Geisser, *P1*, *P* = 0.001). Notably, we found that EEQs are preferentially metabolized into their diols (ratio DiHETE/EEQs at *P1*, 0.356 ± 0.100) compared to EpOMEs, EETs and EDPs (ratios diols/epoxy‐metabolites at *P1*, 0.051 ± 0.013, 0.043 ± 0.007, 0.009 ± 0.004, respectively) (Table 6). The following order of ratios was identified: DiHETEs/EEQs> DHETs/EETs = DiHOMEs/EpOMEs> DiHDPA/EDPs in RBCs (paired *t*‐tests, one‐tailed, Bonferroni correction). In our earlier studies, we observed similar results for plasma diols/epoxides in plasma (Gollasch et al., 2019). The ratios did not change in response to exercise (Table 6). This state‐of‐affairs was also the case for to the individual diol/epoxide metabolites in RBCs (Table 7). In our previous studies, we obtained similar results for plasma diols/epoxides in plasma (Gollasch et al., 2019). The observed findings are unlikely to result from changes in the levels of arachidonic, linoleic acid, docosahexanoic, or eicosapentaenoic acid (Fig. 1) as we previously found that acute, maximal exercise is insufficient to change their levels (Gollasch et al., 2019).

**Table 6:** Ratios estimated using total concentrations of *cis‐*epoxides and diols in erythrocytes in response to exhaustive exercise (*n* = 6).

Epoxides or Diols (ng/g) or Ratios	Time point 1 (rest),* P1*	Time point 2 (HF 150),* P2*	Time point 3 (exhaustion),* P3*	Time point 4 (recovery),* P4*	Greenhouse–Geisser, *P* value
9,10‐EpOME+12,13‐EpOME	200.87 ± 24.05	242.41 ± 28.57	250.19 ± 35.01	257.68 ± 40.38	0.001
9,10‐DiHOME + 12,13‐DiHOME	10.44 ± 4.29	17.36 ± 13.09	11.15 ± 3.02	14.65 ± 7.90	0.255
Ratio (9,10‐DiHOME + 12,13‐DiHOME) / (9,10‐EpOME + 12,13‐EpOME)	0.0507 ± 0.0133	0.0707 ± 0.0534	0.0443 ± 0.0085	0.0561 ± 0.0283	0.335
5,6‐EET + 8,9‐EET + 11,12 EET + 14,15‐EET	277.11 ± 40.02	318.06 ± 33.40	306.68 ± 49.10	323.22 ± 46.52	0.058
5,6‐DHET + 8,9‐DHET + 11,12‐DHET + 14,15‐DHET	11.63 ± 1.39	12.97 ± 1.07	12.16 ± 0.76	12.85 ± 1.47	0.248
Ratio (5,6‐DHET + 8,9‐DHET + 11,12‐DHET + 14,15‐DHET) / (5,6‐EET + 8,9‐EET + 11,12 EET + 14,15‐EET)	0.0427 ± 0.0076	0.0413 ± 0.0062	0.0409 ± 0.0094	0.0404 ± 0.0064	0.763
5,6‐EEQ + 8,9‐EEQ + 11,12‐EEQ + 14,15‐EEQ + 17,18‐EEQ	12.81 ± 4.28	13.19 ± 3.43	13.09 ± 3.18	13.51 ± 4.22	0.814
5,6‐DiHETE + 8,9‐DiHETE + 11,12‐DiHETE + 14,15‐DiHETE + 17,18‐DiHETE	4.28 ± 1.15	4.41 ± 0.76	5.22 ± 0.63	3.98 ± 0.48	0.091
Ratio (5,6‐DiHETE + 8,9‐DiHETE + 14,15‐DiHETE + 17,18‐DiHETE) / (5,6‐EEQ + 8,9‐EEQ + 11,12‐EEQ + 14,15‐EEQ + 17,18‐EEQ)	0.3566 ± 0.1004	0.3622 ± 0.1344	0.4199 ± 0.1104	0.3165 ± 0.1001	0.071
7,8‐EDP + 10,11‐EDP + 13,14‐EDP + 16,17‐EDP + 19,20‐EDP	147.24 ± 24.88	169.77 ± 36.84	160.14 ± 34.18	170.91 ± 37.80	0.057
7,8‐DiHDPA + 10,11‐DiHDPA + 13,14‐DiHDPA + 16,17‐DiHDPA + 19,20‐DiHDPA	1.50 ± 0.77	1.55 ± 0.63	1.68 ± 0.78	1.75 ± 0.62	0.331
Ratio (7,8‐DiHDPA + 10,11‐DiHDPA + 13,14‐DiHDPA + 16,17‐DiHDPA + 19,20‐DiHDPA) / (7,8‐EDP + 10,11‐EDP + 13,14‐EDP + 16,17‐EDP + 19,20‐EDP)	0.0098 ± 0.0042	0.0090 ± 0.0029	0.0101 ± 0.0032	0.0101 ± 0.0024	0.686

**Table 7:** Ratios estimated using individual concentrations of total *cis‐*epoxides and their diols in erythrocytes in response to exhaustive exercise (*n* = 6).

Ratios	Time point 1 (rest),* P1*	Time point 2 (HF 150),* P2*	Time point 3 (exhaustion),* P3*	Time point 4 (recovery),* P4*	Greenhouse–Geisser, *P* value
9,10‐DiHOME / 9,10‐EpOME	0.0320 ± 0.0097	0.0508 ± 0.0460	0.0275 ± 0.0052	0.0364 ± 0.0238	0.319
12,13‐DiHOME / 12,13‐EpOME	0.0698 ± 0.0169	0.0912 ± 0.0606	0.0610 ± 0.0117	0.0755 ± 0.0322	0.345
5,6‐DHET / 5,6‐EET	0.0865 ± 0.0130	0.0818 ± 0.0123	0.0791 ± 0.0146	0.0810 ± 0.0088	0.592
8,9‐DHET / 8,9‐EET	0.0476 ± 0.0098	0.0490 ± 0.0075	0.0491 ± 0.0129	0.0482 ± 0.0101	0.911
11,12‐DHET / 11,12‐EET	0.0282 ± 0.0065	0.0279 ± 0.0052	0.0270 ± 0.0071	0.0261 ± 0.0050	0.618
14,15‐DHET / 14,15‐EET	0.0215 ± 0.0045	0.0190 ± 0.0027	0.0199 ± 0.0053	0.0192 ± 0.0035	0.503
5,6‐DiHETE / 5,6‐EEQ	0.8995 ± 0.6214	0.4716 ± 0.5161	0.6989 ± 0.6652	0.2429 ± 0.5950	0.088
8,9‐DiHETE / 8,9‐EEQ	0	0	0	0	n/a
11,12‐DiHETE / 11,12‐ EEQ	0	0	0	0	n/a
14,15‐DiHETE / 14,15‐EEQ	0	0	0	0	n/a
17,18‐DiHETE / 17,18‐EEQ	0.9919 ± 0.3072	1.1739 ± 0.4890	1.2901 ± 0.3398	1.100 ± 0.2536	0.286
7,8‐DiHDPA / 7,8‐EDP	0.0261 ± 0.0067	0.0235 ± 0.0063	0.0267 ± 0.0058	0.0309 ± 0.0105	0.270
10,11‐DiHDPA / 10,11‐EDP	0.0039 ± 0.0020	0.0038 ± 0.0021	0.0041 ± 0.0023	0.0038 ± 0.0021	0.851
13,14‐DiHDPA / 13,14‐EDP	0.0101 ± 0.0082	0.0085 ± 0.0067	0.0081 ± 0.0063	0.0081 ± 0.0063	0.169
16,17‐DiHDPA / 16,17‐EDP	0.0089 ± 0.0103	0.0083 ± 0.0098	0.0110 ± 0.0089	0.0091 ± 0.0077	0.734
19,20‐DiHDPA / 19,20‐EDP	0.0019 ± 0.0047	0.0022 ± 0.0054	0.0019 ± 0.0046	0	0.606

Together the results indicate that differences in the total RBC levels of 9,10‐EpOME, 12,13‐EpOME, 5,6‐EET, 11,12‐EET, 14,15‐EET), 16,17‐EDP, 19,20‐EDP occur from increased production and accumulation, rather than altered sEH activity during acute, maximal exercise. Since inhibitors of sEH are in clinical development, these findings could be important for patients.

### *Trans*‐epoxides

*Trans*‐EETs are stored in phospholipids and in RBCs in the circulation, and have been proposed to contribute to the antihypertensive effects of sEH inhibitors in rats (Jiang et al., 2011). We measured trans‐epoxy fatty acids in RBCs. We observed that *trans*‐EETs, *trans*‐EEQs, and *trans*‐EDPs are stored at significant levels in human RBCs (Table 8). The total concentrations of these *trans*‐metabolites are ~ 1.5‐2‐fold lower than the concentrations of their respective *cis*‐metabolites (cf. Table 3; Table 9). No changes occurred in the *trans*‐metabolites in response to exercise (Table 8). Similar results were observed for free *trans*‐EETs, *trans*‐EEQs and *trans*‐EDPs (Table 10). Our results suggest that these RBC *trans*‐epoxides presumably do not contribute to the metabolic and hemodynamic response in acute, maximal exercise.

**Table 8:** Total *trans*‐metabolites in erythrocytes in response to exhaustive exercise (*n* = 6).

Metabolite (ng/g)	Time point 1 (rest), *P1*	Time point 2 (HF 150), *P2*	Time point 3 (exhaustion), *P3*	Time point 4 (recovery), *P4*	Greenhouse–Geisser, *P* value
5,6‐EET trans	30.05 ± 11.03	26.83 ± 4.98	30.78 ± 7.34	30.17 ± 6.18	0.386
8,9‐EET trans	44.41 ± 13.64	39.18 ± 7.17	45.11 ± 10.86	48.67 ± 8.84	0.280
11,12‐EET trans	47.38 ± 15.21	44.90 ± 9.08	50.60 ± 12.36	52.31 ± 9.96	0.388
14,15‐EET trans	39.00 ± 12.52	36.16 ± 6.14	41.68 ± 9.19	43.40 ± 7.57	0.296
8,9‐EEQ trans	0	0	0	0	n/a
11,12‐EEQ trans	0.99 ± 0.32	1.05 ± 0.30	1.05 ± 0.22	1.04 ± 0.18	0.887
14,15‐EEQ trans	1.89 ± 0.52	1.84 ± 0.50	2.01 ± 0.53	2.25 ± 0.57	0.088
17,18‐EEQ trans	2.40 ± 0.57	2.43 ± 0.53	2.42 ± 0.73	2.43 ± 0.54	0.99
7,8‐EDP trans	11.59 ± 1.22	10.00 ± 1.55	11.73 ± 2.25	12.44 ± 2.79	0.160
10,11‐EDP trans	33.23 ± 5.52	30.44 ± 5.02	34.73 ± 7.43	36.68 ± 8.70	0.242
13,14‐EDP trans	14.23 ± 1.62	13.66 ± 2.26	15.46 ± 3.26	16.17 ± 4.13	0.267
16,17‐EDP trans	17.03 ± 2.06	15.16 ± 2.17	17.77 ± 4.20	17.98 ± 4.73	0.207
19,20‐EDP trans	13.72 ± 1.94	12.28 ± 1.96	14.09 ± 3.29	15.15 ± 4.17	0.249

**Table 9:** Ratios of total *cis*‐metabolites versus total trans‐metabolites in erythrocytes (*n* = 6).

Metabolite (ng/g)	*Cis*, Time point 1 (rest), *P1*	*Trans*, Time point 1 (rest), *P1*	Ratio (*Cis/Trans*)
5,6‐EET	54.75 ± 6.93	30.05 ± 11.03	1.8
8,9‐EET	70.70 ± 11.08	44.41 ± 13.64	1.6
11,12‐EET	71.69 ± 9.72	47.38 ± 15.21	1.6
14,15‐EET	79.97 ± 13.23	39.00 ± 12.52	2.1
11,12‐EEQ	2.38 ± 0.78	0.99 ± 0.32	2.4
14,15‐EEQ	2.67 ± 0.86	1.89 ± 0.52	1.4
17,18‐EEQ	3.62 ± 0.94	2.40 ± 0.57	1.5
7,8‐EDP	29.72 ± 5.06	11.59 ± 1.22	2.6
10,11‐EDP	42.64 ± 7.31	33.23 ± 5.52	1.3
13,14‐EDP	21.34 ± 4.31	14.23 ± 1.62	1.5
16,17‐EDP	27.04 ± 3.95	17.03 ± 2.06	1.6
19,20‐EDP	26.50 ± 4.83	13.72 ± 1.94	1.9

**Table 10:** Free *trans*‐metabolites in blood plasma in response to exhaustive exercise (*n* = 6).

Metabolite (ng/ml)	Time point 1 (rest),* P1*	Time point 2 (HF 150),* P2*	Time point 3 (exhaustion),* P3*	Time point 4 (recovery),* P4*	Greenhouse–Geisser, *P* value
5,6‐EET trans	2.77 ± 0.67	3.01 ± 1.14	2.74 ± 0.99	2.59 ± 0.67	0.458
8,9‐EET trans	4.43 ± 1.18	4.95 ± 1.55	4.44 ± 1.35	4.15 ± 1.17	0.296
11,12‐EET trans	4.70 ± 1.23	5.05 ± 1.60	4.50 ± 1.52	4.15 ± 1.27	0.306
14,15‐EET trans	3.81 ± 0.97	4.05 ± 1.32	3.69 ± 1.14	3,53 ± 1.16	0.461
8,9‐EEQ trans	0.16 ± 0.07	0.18 ± 0.06	0.20 ± 0.08	0.18 ± 0.07	0.525
11,12‐EEQ trans	0.33 ± 0.08	0.30 ± 0.13	0.32 ± 0.08	0.27 ± 0.10	0.444
14,15‐EEQ trans	0.38 ± 0.08	0.37 ± 0.11	0.35 ± 0.07	0.33 ± 0.12	0.293
17,18‐EEQ trans	0.45 ± 0.09	0.46 ± 0.14	0.45 ± 0.14	0.41 ± 0.12	0.539
7,8‐EDP trans	1.15 ± 0.22	1.30 ± 0.48	1.18 ± 0.43	1.05 ± 0.21	0.328
10,11‐EDP trans	3.36 ± 0.65	3.66 ± 1.47	3.35 ± 1.34	2.92 ± 0.58	0.347
13,14‐EDP trans	1.37 ± 0.58	1.42 ± 0.51	1.28 ± 0.42	1.26 ± 0.21	0.531
16,17‐EDP trans	1.29 ± 0.35	1.35 ± 0.56	1.16 ± 0.51	1.06 ± 0.27	0.138
19,20‐EDP trans	1.34 ± 0.33	1.49 ± 0.55	1.30 ± 0.54	1.22 ± 0.29	0.403

## Discussion

To the best of our knowledge, we conducted the first study on the impact of acute exercise on eicosanoids in RBCs. Epoxy‐metabolite and hydroxy RBC profiling was performed on venous blood taken form healthy individuals. The volunteers were subjected to maximal treadmill exercise using the standard Bruce protocol. This protocol induces strong and robust hemodynamic and metabolic changes (Bruce et al., 1973; Trappe and Lollgen, 2000). We confirmed our hypothesis that individual RBC EETs and EDPs are influenced by acute, exhaustive exercise, but *trans*‐epoxy and LOX metabolites are not. These findings indicate involvement of CYP. These changes were associated with heart rate, systolic blood pressure, and serum lactate increases, which occurred at 13.5 METs. Our results indicate that RBC could represent a reservoir for PUFA CYP epoxy‐metabolites, which on release may act in a vasoregulatory capacity to affect hemodynamics in acute, maximal exercise.

### EETs

RBCs have been implicated to serve as a reservoir for EETs which on release may act in a vasoregulatory capacity (Jiang et al., 2010; Jiang et al., 2011). In addition to serving as carriers of O_2_, RBCs regulate vascular resistance and the distribution of microvascular perfusion by liberating ATP and EETs upon exposure to a low O_2_ environment (Jiang et al., 2010; Sprague, 2010). The release of EETs is activated by ATP stimulation *via* P2X_7_ receptors coupled to ATP transporters to amplify the circulatory response to ATP (Jiang et al., 2007). RBCs are reservoirs of EETs. As a matter of fact, RBC may serve as a primary source of plasma EETs, which are esterified to the phospholipids of lipoproteins. Levels of free EETs in plasma are reduced, namely in the range of ~3% of circulating EETs (Jiang et al., 2010; Jiang et al., 2011). RBC EETs are produced by direct oxidation of arachidonic acid (AA) esterified to glycerophospholipids and the monooxygenase‐like activity of hemoglobin (Jiang et al., 2010; Jiang et al., 2011; Jiang et al., 2012). On release, EETs exert effects on vascular tone and blood pressure. In addition, EETs produce pro‐fibrinolysis and reduce inflammation (Jiang et al., 2010; Jiang et al., 2011; Jiang et al., 2012). An sEH is now recognized to regulate the RBC EET levels by EET catabolism into DHETs ( Jiang et al., 2011). Our results demonstrate that physical exercise affects the RBC CYP epoxy‐metabolite status, which could contribute to the hemodynamic and metabolic response in acute, exhausting exercise. In particular, we observed increases in total concentrations of EETs (5,6‐EET, 11,12‐EET, 14,15‐EET) in RBCs, which are potent vasodilators (Jiang et al., 2010; Sprague, 2010; Jiang et al., 2011). We did not observe an increase in RBC DHETs levels. DHETs were initially thought to be inactivation products of EETs. Nonetheless, recent studies indicate that, like EETs, they produce vasodilation (Hercule et al., 2009), possibly by activating BK_Ca_ channels in vascular smooth muscle cells (Lu et al., 2001). It is unknown whether RBCs are capable of liberating DHETs upon exposure to a low O_2_ environment or ATP. Our results indicate that short‐term maximal exercise affects the RBC reservoir for EETs, but not DHETs, which on release may act in a vasoregulatory capacity to affect the cardiovascular response.

### Other PUFA metabolites

We observed increases in total concentrations of EDPs (16,17‐EDP, 19,20‐EDP) and EpOMEs (9,10‐EpOME and 12,13‐EpOME) in RBCs during maximal exercise. 16,17‐EDP and 19,20‐EDP are potent vasodilators in coronary, pulmonary and mesenteric arteries and can lower blood pressure and exhibit cardioprotection by preservation of mitochondrial function (Morin et al., 2011; Schunck et al., 2017). In contrast, 9,10‐EpOME and 12,13‐EpOME are reported to represent vasoconstrictors in severe cardiac ischemia (Siegfried et al., 1990; Dudda et al., 1996). However, it is unknown whether RBCs are capable of liberating EDPs or EpOMEs upon exposure to a low O_2_ environment or ATP. Our data indicate that both metabolites are novel candidates for vasoactive substances potentially released by RBCs upon maximal exercise to affect hemodynamics in these conditions.

Furthermore, we observed that total levels of the CYP metabolites 5,6‐EEQ, 8,9‐EEQ, 11,12‐EEQ, 14,15‐EEQ, 8,9‐EET, 10,11‐EDP, and 13,14‐EDP in RBCs were not influenced by acute, exhaustive exercise. Similar effects were observed for the LOX metabolites 5‐HEPE, 8‐HEPE, 9‐HEPE, 12‐HEPE, 15‐HEPE, 18‐HEPE, 5‐HETE, 8‐HETE, 9‐HETE, 11‐HETE, 12‐HETE, 15‐HETE, 4‐HDHA, 7‐HDHA, 8‐HDHA, 10‐HDHA, 11‐HDHA, 13‐HDHA, 14‐HDHA, 16‐HDHA, 17‐HDHA, 20‐HDHA, and 13‐HODE. EEQs levels were low because the subjects did not receive long chain n‐3 fatty acid supplementation. The steadiness of the HDHAs and HEPEs is of particular interest because these DHA and EPA‐derived metabolites are the precursors of the D‐ and E‐series of resolvins. Resolvins are members of a novel class of lipid mediators that have highly potent antiinflammatory and pro‐resolution properties (Fischer et al., 2014). Together, the data suggest that these PUFA metabolites are unlikely to play a role in the cardiovascular response caused by short‐term exercise.

### Diols

sEH converts EETs to DHETs, EpOMEs to DiHOMEs, EEQs to DiHETEs, EDPs to DiHDPAs (Fig. 1) and inhibition of sEH is a potential approach for enhancing the biological activity of EETs (Spector and Kim, 2015). However, presumably higher levels of EETs in blood and tissue *in vivo* may have also detrimental cardiovascular side effects, such as increased risk of ischemic stroke (Gschwendtner et al., 2008) and higher recurrence of atrial fibrillation after catheter ablation in humans (Wutzler et al., 2013), and reduced survival after cardiac arrest and cardiopulmonary resuscitation in mice (Hutchens et al., 2008). We cannot exclude that the higher levels of EETs, EDPs, and EPOMEs in RBCs during maximal exercise result from sEH inhibition. However, this is rather unlikely since our ratio analyses argue against this possibility. Furthermore, the DHET, DiHOME and DiHDPA levels did also not vary during acute, exhausting exercise in healthy volunteers. Finally, short‐term exhaustive exercise did not cause increased mobilization of individual free epoxy‐metabolites into RBC plasma. Nevertheless, the effects of the erythro‐DHETs, erythro‐DiHOMEs, erythro‐DiHDPAs has yet to be integrated into a patho‐physiological context.

### Epoxy‐metabolites in the recovery period

Accumulation of total 5,6‐EET, 11,12‐EET, 14,15‐EET, 16,17‐EDP, 19,20‐EDP, 9,10‐EpOME, and 12,13‐EpOME levels in RBCs persisted in the recovery period (10 min). The reason is unclear but may represent a phenomenon similar to the recovery pulse sum, which becomes larger when the loading intensity is above the long‐term performance limit. We suspect that levels of 5,6‐EET, 11,12‐EET, 14,15‐EET, 16,17‐EDP, 19,20‐EDP, 9,10‐EpOME, and 11,12‐EpOME in RBCs will return to normal levels after a longer resting time interval.

### Exercise protocol considerations

The Bruce‐protocol was used to ensure that all runners were able to complete a similar highest intensity workload concomitant with robust and significant increases in hemodynamics without fatiguing. Our clinical data (Table 2) show that the protocol caused the expected hemodynamic and metabolic responses.

### Limitations

Our treadmill testing was primarily developed to identify ischemic heart disease; fortunately, none of our subjects had this condition. Other exhaustive protocols might have been better. Furthermore, the workload was increased in large increments. Primarily the leg musculature was stressed. We obtained venous blood samples and perhaps a source closer to contracting muscle would have been more elucidative (Giordano et al., 2011). We studied effects of maximal short‐term exercise and therefore cannot comment on endurance exercise. Possibly, different results would have been obtained.

## Conclusions

Our results suggest that maximal exercise affects the levels of numerous CYP epoxy‐metabolites in RBCs. We suggest that RBCs are reservoir for EETs and possibly other epoxide fatty acids, which on release may act in a vasoregulatory capacity. More research is needed to delineate the contribution of RBC epoxy‐metabolites to cardiac performance and skeletal‐muscle blood flow in health and disease.

## Conflict of Interest

None.
